# Transcriptional Control of Synaptic Plasticity by Transcription Factor NF-*κ*B

**DOI:** 10.1155/2016/7027949

**Published:** 2016-01-06

**Authors:** Christian Engelmann, Ronny Haenold

**Affiliations:** Leibniz Institute on Aging-Fritz Lipmann Institute (FLI), Beutenbergstrasse 11, 07745 Jena, Germany

## Abstract

Activation of nuclear factor kappa B (NF-*κ*B) transcription factors is required for the induction of synaptic plasticity and memory formation. All components of this signaling pathway are localized at synapses, and transcriptionally active NF-*κ*B dimers move to the nucleus to translate synaptic signals into altered gene expression. Neuron-specific inhibition results in altered connectivity of excitatory and inhibitory synapses and functionally in selective learning deficits. Recent research on transgenic mice with impaired or hyperactivated NF-*κ*B gave important insights into plasticity-related target gene expression that is regulated by NF-*κ*B. In this minireview, we update the available data on the role of this transcription factor for learning and memory formation and comment on cross-sectional activation of NF-*κ*B in the aged and diseased brain that may directly or indirectly affect *κ*B-dependent transcription of synaptic genes.

## 1. Introduction

Acquisition and consolidation of new information by neuronal networks often referred to as learning and memory formation depend on the instant alterations of electrophysiological parameters of synaptic connections (long-term potentiation, long-term depression), on the generation of new neurons (neuroneogenesis), on the outgrowth of axons and dendrites (neuritogenesis), and on the formation/remodulation of dendritic spines (synaptogenesis). The transmission of active synapses becomes potentiated by additional opening of calcium channels and incorporation of preexisting channel proteins, that is, during the induction of long-term potentiation. In contrast, long-term structural reorganization of the neuronal network depends on the induction of specific gene expression programs [1]. The transcription factor NF-*κ*B has been shown to be involved in all of the aforementioned processes of learning-associated neuronal plasticity, that is, long-term potentiation, neuroneogenesis, neuritogenesis, and synaptogenesis (for review, see [2]). With respect to the diverse functions of NF-*κ*B in neuroneogenesis and neuritogenesis, and to its local function as structural protein at the postsynaptic membrane, we refer to specialized review articles [3, 4].

**Figure 1:**
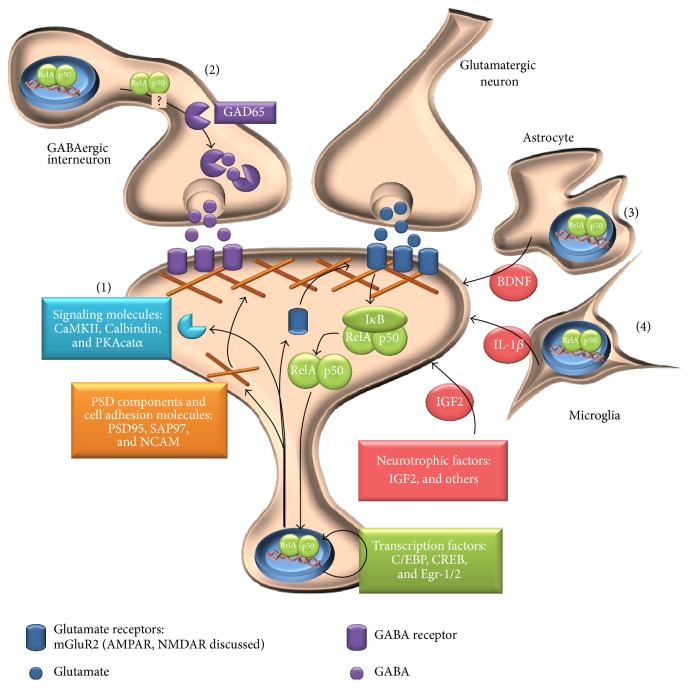
Overview of NF-*κ*B regulated components in synaptic plasticity. (1) At the postsynapse NF-*κ*B regulates the expression of various components of the postsynaptic density, ion channels/receptors, signaling molecules, and transcription factors that are involved in the modulation of synaptic transmission. (2) In inhibitory neurons, NF-*κ*B regulates the expression of GAD65 that is required for GABA synthesis. (3) Synaptic plasticity is further modulated by paracrine release of neurotrophic factors from adjacent cells. In astrocytes, activation of NF-*κ*B led to the secretion of BNDF, and (4) microglia express the NF-*κ*B target gene IL-1*β*. For references, see Table 1.

In mammals, NF-*κ*B consists of five subunits (RelA, RelB, c-Rel, p105/50, and p100/52), possessing either transcriptional activator (Rel proteins) or repressor (p50, p52) functions. Within the CNS, NF-*κ*B signaling encompasses activation of mainly RelA, c-Rel, and p50 containing heterodimers (canonical pathway). In addition to an inducible form in neurons and glial cell populations where NF-*κ*B becomes rapidly activated under metabolic or traumatic stress [5, 6], the transcription factor also possesses constitutive activity in subsets of neuronal cell populations [7]. This has been convincingly presented in brains of *κB-lacZ* reporter mice that express the enzyme beta-galactosidase in dependence on NF-*κ*B transcriptional activation [8, 9]. It has been generally assumed that spontaneous NF-*κ*B activation in the absence of any obvious stimulation exerts a function for neuronal development and maintenance of the mature CNS. However, converse studies on primary neurons, in which such an activation was nearly absent [10], evoke questions on unknown physiological activation mechanisms under steady-state-conditions. One reasonable explanation for the aforementioned constitutive NF-*κ*B activity lies in a synaptic plasticity associated activation that occurs during basal neuronal function [11]. Such activity-dependent activation is supported by a number of observations and facts leading to the conclusion that NF-*κ*B signaling is directly involved in spinogenesis and strengthening of synaptic connections during learning and memory formation (Figure 1).

*(i) All NF-κB Pathway Proteins Are Present at the Synapse*. This has been shown by immunochemistry and biochemical analysis of isolated synaptosomes (for recent review, see [12]). Within the synaptosome, NF-*κ*B is localized to a free synaptoplasmic pool and a membrane-anchored pool, with a dynamic exchange between both pools. This further led to the hypothesis for a local role of NF-*κ*B at the synapse in addition to its nuclear function as a transcription factor, that is, for labeling and preparing the synapse for remodeling and plastic changes [13].

On the subcellular level, NF-*κ*B is concentrated at the postsynaptic density and dendritic cytoplasm to induce gene expression within the signal-receiving neuron. Immunohistochemical analysis of brain sections revealed also an axonal localization of NF-*κ*B proteins* in vivo* [14, 15]. Although the functional relevance of such axoplasmic NF-*κ*B requires further investigation, it points to a presynaptic feedback mechanism that might respond to synaptic stimulation by retrogradely transported NF-*κ*B. For example, NF-*κ*B regulates presynaptic transmitter production of *γ*-aminobutyric acid releasing (GABAergic) interneurons by direct or indirect transcriptional upregulation of the GABA synthetizing enzyme glutamate decarboxylase 65 (GAD65) [16]. Thereby, changes in GABAergic NF-*κ*B activity can affect the magnitude of inhibitory transmission. Functionally, transgenic mice with overexpression of the NF-*κ*B superrepressor I*κ*B*α*-SR in GABAergic neurons display enhanced excitatory signal transduction, increased long-term potentiation, and enhanced spatial learning and memory [16]. Overall, presynaptic functions of *κ*B-dependent gene expression for synaptic plasticity require further investigations.

*(ii) NF-κB Becomes Activated at Active Synapses*. According to its function as a synapse-to-nucleus signal transducer in the postsynaptic neuron NF-*κ*B is locally activated by synaptic transmission and retrogradely transported into the neuronal soma. Stimulation of various receptors leads to the activation of NF-*κ*B within the postsynaptic compartment (for review, see [17]). One mechanism that has been described in detail involves the activation of group I metabotropic glutamate receptors (GpI-mGluRs) in excitatory neurons. Stimulation of hippocampal neurons with a GpI-mGluR agonist results in nuclear translocation of NF-*κ*B within one hour as detected by a temporary increase in DNA binding activity of p50, RelA, and c-Rel [18]. Three major signaling pathways involving protein kinase C (PKC), calmodulin-derived, and Ras/PI3K/Akt cascades link postsynaptic receptor stimulation to local phosphorylation of IKK kinases, which represents the key regulatory step for NF-*κ*B activation. They all involve the opening of calcium channels at the plasma membrane and intracellular calcium stores to increase synaptoplasmic calcium levels, which represents a specific feature of local NF-*κ*B activation at neuronal synapses [19]. Degradation of inhibitory I*κ*B*α* mobilizes the NF-*κ*B dimer and exposes the nuclear localization sequence, which is required for its dynein/dynactin-dependent transport along microtubules into the nucleus [12, 20]. The exact dynamics of subcellular redistribution of NF-*κ*B from the synaptosomal cytoplasm and membrane to the nucleus during memory consolidation is currently a matter of investigation [13].

*(iii) NF-κB Induces Expression of Target Genes for Synaptic Plasticity*. Studies on knockout mice have facilitated the search for NF-*κ*B target genes linked to synaptic plasticity. One of the first genes identified is the *α* catalytic subunit of protein kinase A (PKAcat*α*) whose expression is pivotal for the induction of synaptic plasticity and spatial learning in mice. The promoter region of the* PKAcatα* gene contains one NF-*κ*B binding site that is conserved in several species and binding of RelA/p50 to this site has been demonstrated by band shift assays, thus indicating a direct transcriptional regulation of PKAcat*α* by NF-*κ*B [21]. There is a growing list of genes that are induced by NF-*κ*B in the context of synaptic plasticity albeit the direct or indirect transcriptional regulation mechanism has not always been determined (for recent review, see [22]). These targets represent a wide range of functions such as scaffolding and cell adhesion proteins, neurotrophic factors, neurotransmitters, ion channels, and signaling molecules (Table 1). The identification of further NF-*κ*B target genes and the exploration of their subunit and context-specific upregulation during different learning paradigms will be the real challenge to fully understand the diversity of target genes orchestrated by NF-*κ*B. Moreover, there is growing evidence for the requirement of *κ*B-dependent gene expression in nonneuronal cells that locally support synaptic plasticity (Figure 1). For example, inhibition of NF-*κ*B specifically in astrocytes by overexpressing a dominant-negative form of I*κ*B*α* (*GFAP-IκBα-dn*) impaired spatial and nonspatial learning in female mice [23]. This was accompanied by a reduced expression of neuron-specific PSD95 and mGluR5. Despite the fact that astrocyte-specific target genes of NF-*κ*B have not been investigated in this study, the data strongly suggest that astrocytes positively modulate the expression of synaptic proteins by neurons. To investigate the contribution of microglial NF-*κ*B to learning and memory formation in mice, IKK2 has been deleted in myeloid cells including microglia (*mIKK2KO*) recently [24]. Among various well-known target genes of NF-*κ*B only transcript levels for* Interleukin-1β* were altered in the brains of* mIKK2KO* mice, which coincided with transiently increased short-term fear memory of the transgenic mice. This observation reveals a novel and unexpected role for microglial IKK2/NF-*κ*B in the homeostatic regulation of synaptic plasticity [24]. Taken together, synaptic plasticity requires the adaptive regulation of *κ*B-responsive genes not only within the pre- and postsynaptic neuron, but also within adjacent astro- and microglial cells.

*(iv) Activation of NF-κB Is Required for Learning and Memory Formation*. Notably, NF-*κ*B signaling is obviously dispensable for normal CNS development. This has been shown in a number of mouse lines with congenital impaired NF-*κ*B signaling. Transgenic mice with CNS-specific deletion of abundantly expressed RelA or inactivated upstream regulators of NF-*κ*B (I*κ*B*α*, IKK) in the neuroglial compartment are indiscernible regarding overall neuroanatomical and behavioral features [5, 6, 25, 26]. However, a large number of behavioral studies on animals with inactivated NF-*κ*B provide convincing evidence for its requirement specifically in learning and memory formation. These experiments have been performed on different species (crab [27], mouse [28], and rat [29]), with different approaches for NF-*κ*B inactivation (pharmacological, NF-*κ*B decoy, and genetic knockout) and by testing different forms of learning conditions (long-term habituation, fear conditioning, and spatial learning) (for review on evolutionarily conserved roles in synaptic plasticity, see [22, 30]). Altogether, they implicate that among the subunits investigated (p50, RelA, and c-Rel) all are required for the establishment of learning and memory formation. However, it has to be stated that the specific learning mechanisms in these species, that is, synaptic plasticity, have not always been explicitly investigated. Moreover, there exists only very limited data on systematic knockout studies that allow for conclusions on NF-*κ*B subunits in individual tests. Recently, we have addressed this question by studying cortical plasticity of monocular deprived p50 knockout (*p*50^*KO*^) mice and mice with CNS-restricted deletion of RelA (*RelA* ^*CNSKO*^). The results show a nonredundant requirement of both subunits of the classical NF-*κ*B pathway, RelA, and p50, for the establishment of synaptic plasticity (unpublished data). Functionally, this obligation stands in contrast to the injury-induced activation of NF-*κ*B in the lesioned brain, such as after stroke and axonal fiber injury. Here, antagonistic effects of the transactivator subunit RelA and the transcriptional repressor p50 for neuronal survival have been observed [5, 6].

The aforementioned results implicate that canonical NF-*κ*B acts as a positive regulator of synaptic plasticity by transcriptional upregulation of synaptic proteins. In the past, this role of NF-*κ*B has been addressed almost exclusively using loss-of-function mutants with deletion of specific NF-*κ*B subunits (p50, c-Rel, and RelA), or by pan-inhibition of NF-*κ*B following overexpression of inactive IKK or nondegradable forms of the NF-*κ*B inhibitor I*κ*B*α*. Recently, studies on NF-*κ*B gain-of-function mutants have been performed providing additional and exciting insights into the pivotal role of NF-*κ*B as an enhancer for synaptic plasticity thereby demonstrating its potential for clinical applications. According to its proposed function as a transcriptional activator of activity-dependent gene expression genetically induced hyperactivation of NF-*κ*B signaling should maximize synaptic plasticity by reinforcing *κ*B-dependent gene expression of synaptic proteins. This assumption has been tested in mice with disinhibited NF-*κ*B signaling in which autoinhibitory upregulation of I*κ*B*α* is impaired by the mutation of the* ikba* promoter (*IκBα* ^*M*/*M*^) [31]. Primary neurons from *IκBα* ^*M*/*M*^ mice stimulated with TNF displayed a sustained NF-*κ*B activation compared to WT neurons. During neuronal culture dissociated hippocampal neurons form excitatory and inhibitory synaptic contacts that can be stained with antibodies against the glutamatergic presynaptic marker VGLUT or the GABAergic presynaptic marker VGAT. Quantitative analysis of the synaptic puncta revealed an increase in excitatory synapse density and a decrease in inhibitory synapse density on transgenic neurites suggesting an altered synaptic connectivity within *IκBα* ^*M*/*M*^ hippocampal neurons [31]. Functionally, the imbalance of glutamatergic and GABAergic synaptic transmission leads to spontaneous burst firing and hyperexcitability of *IκBα* ^*M*/*M*^ neurons. Notably, when tested for learning and memory formation young adult *IκBα* ^*M*/*M*^ mice performed better for hippocampus-dependent contextual fear memory as compared to their littermate controls. The cognitive enhancement in *IκBα* ^*M*/*M*^ mice was further confirmed in the acquisition of spatial memory. Here, *IκBα* ^*M*/*M*^ mice revealed an enhanced retention of the memory. The cognitive improvements occurred independent of any changes in general behavior; that is, the transgenic mice showed normal levels of activity, anxiety-like behavior, or nociception [31].

Another study examined the synaptogenetic effect of hyperactivated NF-*κ*B in a model of drug-addicted behavioral sensitization in mice. First, mice received a viral-mediated gene transfer to express a constitutively active IKK (*IKKca*) in the nucleus accumbens (NA) [32]. Then, they were administered with chronic cocaine, which induced dendritic spine changes. Despite the fact that the rewarding behavior of cocaine-sensitized* IKKca* mice was not changed as tested by conditional place preference (CPP) training, the number of dendritic spines on NA neurons was significantly increased under constitutive activation of NF-*κ*B by* IKKca*. Among the tested factors with known potential for spinogenesis, only BDNF, but not GDNF, was enhanced in* IKKca* mice, suggesting that* bdnf* is a transcriptional target of NF-*κ*B in the NA. Recently, the fibroblast growth factor homologous factor 1 (FHF1/FGF12) has been identified as a physiological “break” of NF-*κ*B activity that restricts neurite and spine formation in mature cortical neurons.* Fhf1* gene silencing strongly activated neuronal NF-*κ*B activity and, thereby, significantly increased spine densities in a NEMO-dependent manner [33]. These results certainly warrant behavioral studies on FHF1-deficient mice in the future. Taken together, these experiments show that hyperactivation of NF-*κ*B can amplify synaptic plasticity by promoting *κ*B-dependent expression of the aforementioned structural, signaling, or neurotrophic factors.

*(v) Open Questions and Future Directions*. While there is convincing evidence for a role of NF-*κ*B in synaptic plasticity, a number of crucial questions remain to be answered. First, only a limited number of studies have examined subunit-specific transcriptional activator and repressor functions of NF-*κ*B. With an increasing list of *κ*B-regulated target genes expressed during synaptic plasticity, this might shed light on the gene-regulatory mechanisms required for the selected expression. Along this line, the role of RelB-dependent NF-*κ*B signaling for synaptic plasticity might be underestimated. Schmeisser et al. presented evidence for robust presence of RelB protein particularly at the synapse by analyzing crude homogenates, synaptosomes, and PSD fraction from adult mouse forebrain for the subcellular localization of NF-*κ*B family members. Interestingly enough, among the Rel proteins only RelB showed an enrichment in the synaptosomal fraction, in contrast to equally distributed RelA or c-Rel, which was almost exclusively present in the PSD [34]. Future studies are required to address synaptogenetic functions of alternative NF-*κ*B signaling.

Second, the influence of NF-*κ*B as a mediator of cellular stress, inflammation, and neurosenescence on *κ*B-dependent gene expression in synaptic plasticity warrants further investigations. Dysregulation of NF-*κ*B signaling by inflammation or environmental factors interferes with neurogenesis in both the developing and the adult brains. For example, cell-intrinsic activation of NF-*κ*B in neural stem cells by acute stress suppresses hippocampal neurogenesis in adult rats [35]. Likewise, maternal infection during pregnancy can affect foetal brain development via the release of proinflammatory activators of NF-*κ*B [36, 37], which might cause neurological disorders like depression and learning disabilities. Finally, constitutive activation of NF-*κ*B is a hallmark of aging leading to its hyperactivation and proinflammatory gene expression in neurons [38, 39]. Indeed, the NF-*κ*B motif has been identified as the most upregulated gene expression program in aged tissues including the brain [40]. It seems plausible that such changes in NF-*κ*B activity might directly affect *κ*B-dependent transcription of synaptic genes and, thus, might contribute to the impairments in synaptic plasticity observed in the aging brain. Vice versa, lifelong attempts in learning and memory formation, which have been demonstrated as a protective factor against premature cognitive impairments, might imply selective activation patterns of NF-*κ*B. Given their high social relevance, the regulation of synaptic plasticity and learning and memory formation by NF-*κ*B warrants further investigations.

## Figures and Tables

**Table 1:** Overview of NF-*κ*B target genes in synaptic plasticity.

Target gene	Location	Effect	Gene description	Reference
Neurotrophic factors				
IGF2	Postsynaptic	Direct	Insulin-like growth factor 2	[34]
BDNF	In astrocytes	Direct	Brain-derived neurotrophic factor	[41]

Structural/adhesion/scaffolding proteins				
PSD95	Postsynaptic	Indirect	Postsynaptic density compartment (also SAP90) membrane-associated guanylate kinase	[23, 34]
SAP97	Postsynaptic	Indirect	Synapse-associated protein 97 or disks large homolog 1 (DLG1)	[34]
NCAM	Postsynaptic	Direct	Neuronal cell adhesion molecule	[42]
ICAM3	Postsynaptic	Direct	Intercellular adhesion molecule 3	[43]
Slitrk1	Postsynaptic	Direct	SLIT and NTRK-like family member 1	[44]
Tiam1	Postsynaptic	Direct	T-cell lymphoma invasion and metastasis-inducing protein 1	[44]

Receptor and signaling proteins				
Calbindin	Postsynaptic	Indirect	Calcium binding protein -D28k and -D9k	[45, 46]
CaMKII *δ*	Postsynaptic	Direct	Ca(2+)/calmodulin-dependent protein kinase type II alpha chain	[47]
CREB	Postsynaptic	Indirect	cAMP response element-binding protein	[21]
C/EBP	Postsynaptic	Direct	CCAAT/enhancer-binding protein transcription factor	[48–50]
Egr-1	Postsynaptic	Direct	Early growth response protein 1 or NGFI-A (nerve growth factor-induced protein A)	[51]
Egr-2	Postsynaptic	Direct	Early growth response protein 2	[52]
Fos	Postsynaptic	c-Rel binding sites identified	Transcription factor	[53, 54]
GAD65	Presynaptic	Indirect	Glutamic acid decarboxylase function in GABA synthesis	[16]
NMDA1 receptor subunit 1 (Grin1)	Postsynaptic	*κ*B binding sites identified	Subunit 1 of N-methyl-D-aspartate glutamate receptor	[55]
NMDA2A receptor subunit 2A (Grin2A)	Postsynaptic	*κ*B binding sites identified	Subunit 2A of N-methyl-D-aspartate glutamate receptor	[56]
mGluR2	Postsynaptic	Direct	Metabotropic glutamate receptor 2	[57]
PKAcat*α*	Postsynaptic (hippocampus)	Direct	Protein kinase A catalytic subunit *α*	[21]

## Abbreviations

BDNF: Brain-derived neurotrophic factor
CNS: Central nervous system
FHF1/FGF12: Fibroblast growth factor homologous factor 1
GABA: *γ*-Aminobutyric acid
I*κ*B*α*-dn: Dominant-negative I*κ*B*α*
I*κ*B*α*-SR: I*κ*B*α* superrepressor
IKK: I*κ*B*α* kinase complex
mGluR5: Metabotropic glutamate receptor type 5
NF-*κ*B: Nuclear factor kappa B
PKAcat*α*: *α* Catalytic subunit of protein kinase A
PSD95: Postsynaptic density protein 95
VGAT: Vesicular GABA transporter
VGLUT: Vesicular glutamate transporter
WT: Wild type.
